# Assessment of the Psychophysiological State of Female Operators Under Simulated Microgravity

**DOI:** 10.3389/fphys.2021.751016

**Published:** 2022-02-11

**Authors:** Svetlana Lebedeva, Dmitry Shved, Alexandra Savinkina

**Affiliations:** ^1^Russian Federation State Scientific Center, Institute of Biomedical Problems of the Russian Academy of Sciences, Moscow, Russia; ^2^Moscow Aviation Institute, National Research University, Moscow, Russia

**Keywords:** dry immersion, women, ground-based model of microgravity, NAIAD-2020, human operator performance, psychophysiological state, speech analysis

## Abstract

The article describes methods of non-verbal speech characteristics analysis used to determine psychophysiological state of female subjects under simulated microgravity conditions (“dry” immersion, DI), as well as the results of the study. A number of indicators of the acute period of adaptation to microgravity conditions was described. The acute adaptation period in female subjects began earlier (evening of the 1st day of DI) and ended faster than in male ones in previous studies (2nd day of DI). This was indicated by a decrease in the level of state anxiety (STAI, *p* < 0,05) and depression-dejection [Profile of Mood States (POMS), *p* < 0,05], as well as a decrease in pitch (*p* < 0,05) and voice intensity (*p* < 0,05). In addition, women, apparently, used the “freeze” coping strategy – the proportion of neutral facial expressions on the most intense days of the experiment was at maximum. The subjects in this experiment assessed their feelings and emotions better, giving more accurate answers in self-assessment questionnaires, but at the same time tried to look and sound as calm and confident as possible, controlling their expressions. Same trends in the subjects’ cognitive performance were identified as in similar experimental conditions earlier: the subjects’ psychophysiological excitement corresponded to better performance in sensorimotor tasks. The difference was in the speed of mathematical computation: women in the present study performed the computation faster on the same days when they made fewer pauses in speech, while in men in previous experiments this relationship was inverse.

## Introduction

In the context of the possibility of long-term flights into deep space, new approaches are currently being developed to create systems for psychophysiological support of the crew. Among the main requirements for such systems there are increased autonomy and minimal invasiveness (Egorov, 2001; Cermack, 2006). Such methods would allow for continuous monitoring of the crew members’ condition, without interfering with their regular activities and with minimal involvement of Mission control specialists.

One of the methods that meets these criteria is automated analysis of psychologically and psychophysiologically relevant characteristics of human operators’ speech (Johannes et al., 2000; Alberdi et al., 2016; Gushin et al., 2016).

Nevertheless, speech is studied mainly using content analysis, which limits the possibility of correct interpretation of the subject’s emotional and psychophysiological state (Nikonov, 1985). On the other hand, the acoustic characteristics of speech are less susceptible to conscious control and better reflect the deep features of the human functional state. For this reason, they have been used for a long time in studies devoted to assessing the functional state of a person in extreme conditions (Kartavenko, 2005).

Previously, the study of the speech acoustic characteristics was successfully carried out in a number of analog and space experiments (Johannes et al., 2008; Gushin et al., 2016; Gushchin et al., 2018; Supolkina et al., 2019). The technological and methodological progress over the years allows the researchers to bring their studies to a new level within the framework of the modern approach, which ultimately presupposes minimizing the subjectivity and invasiveness of psychophysiological and psychological techniques.

At the same time, acoustic analysis of speech often shows reproducibility limitations due to varying experimental conditions. One of the most studied characteristics of speech, fundamental frequency (F0), usually increases when a person experiences physiological or psychological stress (Giddens et al., 2013). However, in a number of studies this was not confirmed. These discrepancies may be associated with different recording conditions, nature of the stress factors, and the individual characteristics of the subjects. Thus, in order to minimize the differences caused by various simulated factors of space flight as well as other possible interfering factors, it is reasonable to analyze the voices of test subjects under the same experimental conditions (National Aeronautics and Space Administration and the National Center for Gender Physiology and Environmental Adaptation, 2002; Park and Stepp, 2019).

The “dry” immersion is currently recognized as the best model for long-term simulation of weightlessness and support-free conditions (Mikhailov et al., 1995; Tomilovskaya et al., 2019). Thus, the conditions of “dry” immersion make it possible to study the psychophysiological state of the subjects in the conditions closest to a real space flight.

The technology and experimental approach have been known and applied since the 1970s, but until the year 2020, only male subjects participated (Shul’zhenko and Vill-Villiams, 1976; Orlov, 1985). Taking into account the underrepresentation of women in space, as well as the small number of ground-based simulation experiments with their participation (National Aeronautics and Space Administration and the National Center for Gender Physiology and Environmental Adaptation, 2002; Saralyn, 2006), it was necessary to start experimental studies with women under conditions of “dry” immersion in order to assess not only the physiological reactions of adaptation to microgravity conditions, but also the psychophysiological response to this type of stress.

The aim of this study was to assess the female subjects’ psychophysiological response to physiological stress caused by adaptation to the conditions of simulated microgravity, and also to determine whether it differs from the analogous response in male subjects in the same conditions.

## Materials and Methods

### Participants

The study involved 6 healthy female volunteers aged 24 to 39 years. The studies were carried out in the same periods of the participants’ natural menstrual cycle (7th–10th days of MC).

### Bioethics and Informed Consent

The conducted studies were approved by the Bioethical Commission of the Institute of Biomedical Problems of RAS (Protocol No. 544 of July 16, 2020) and fully complied with the principles of the 1964 Declaration of Helsinki. Each study participant voluntarily signed an informed consent after explaining to her the potential risks, benefits and nature of the upcoming study.

### Design of the Study

From September 7 to November 30, 2020, the IBMP RAS conducted the world’s first “dry” immersion experiment with the participation of female volunteers («NAIAD-2020») (Tomilovskaya et al., 2021).

The method used in our study is based both on the methodological approaches implemented in previous space and analog experiments, and on recent methods and technologies of speech acoustic analysis.

The main material for the analysis were audio recordings of the subjects’ speech during the morning and evening reports on daily events, health and mood (analogous to daily planning conferences, or DPCs, the ISS-MCC standard communication procedure). The reports were recorded right after waking up (8–9 AM) and before going to sleep (9–10 PM). Portable voice recorders Zoom H1 were used, positioned at 15 cm from the subject’s mouth during the recording.

Simultaneously with the audio recording of DPC audio messages, for the first time, within the framework of the experiment in the conditions of “dry” immersion, video recording was included to our experimental protocol for the analysis of basic emotions using facial expressions (Sanchez et al., 2014). Within the united methodical approach, acoustic analysis of speech and analysis of facial expressions can become promising means of remote monitoring of the psychoemotional and psychophysiological state of human operator both in space flight and in other extreme conditions (Fontes and Madureira, 2019; Gushchin et al., 2020).

We studied only the period of acute adaptation in order to reveal the differences between the female and male psychophysiological responses to adaptation to microgravity conditions. We also used additional cognitive and sensorimotor tests, facial expression assessment using the FaceReader software, Spielberger’ state-trait anxiety inventory (STAI) and Profile of Mood States (POMS), as well as medical control data: systolic (SBP) and diastolic (DBP) blood pressure, and heart rate (HR) provided by Dr. E.S. Tomilovskaya.

### Speech Acoustic Characteristics Analysis

Within the framework of this study, the main method was the analysis of the acoustic characteristics of speech reflecting the functional state of the subjects, with the Praat software (Boersma and Weenink, 2018). We analyzed speech segments unified by the audio reporting protocol, in order to minimize the impact of varying content of statements. The speech fundamental frequency (F0) (Mean and Median pitch values, as calculated by the Praat software algorithm within every acoustic sample), intensity (speech volume), the number of voice pulses and pauses in speech, shimmer and jitter were studied in dynamics over the course of immersion. These indicators are often used as correlates of emotional and psychophysiological stress and pathological conditions in humans, as well as to describe a person’s individual speech “portrait” in order to more reliably identify individual differences in stress responses (Lebedeva et al., 2020; Lebedeva and Shved, 2021).

One of the first still the most studied parameters of speech signal is the pitch (fundamental frequency, F0) (Buchanan et al., 2014; Pisanski et al., 2018). Its measurement made it possible to solve a number of practical problems in speech technologies development, such as the speaker’s gender identification, determining their emotional state, speech recognition, as well as identifying the functional state of the vocal apparatus (Johannes et al., 2007; Teixeira et al., 2011; Alberdi et al., 2016; Akçay and Oguz, 2020).

When measuring the intensity (speech volume), we took into account that in calmly speaking subjects its average value is 40–60 dB, and in an excited state – 70–80 dB. Changes in the intensity are an important diagnostic feature in assessing the psychophysiological state of a person (Rothkrantz, 2004; Zhang, 2016).

Unvoiced fragments length is an absolute value corresponding to the physical duration of the pauses, i.e., a break in sound when the average sound pressure drops to zero at the junction between two sound segments. In relation to the duration of the utterance, the percentage of pauses that a person makes between words and phrases is calculated. This value may indicate a state of psychophysiological tension or asthenization (Buchanan et al., 2014).

The use of jitter and shimmer effects is important for determining the voice pathologies and is often used in the clinical studies (Zwetsch et al., 2006; Teixeira et al., 2011). Jitter is a measure of period-to-period fluctuations in F0, showing involuntary changes in the frequency of adjacent vibrational cycles of the vocal folds. Shimmer is a measure similar to jitter, which characterizes the period-to-period variability of the amplitude value. The reason for the widespread use of jitter and shimmer parameters in clinical studies is that the structure of vibrations of the vocal folds has a periodic nature for healthy voices, while this periodicity in the structure of vibrations is significantly impaired in the presence of pathological changes (Teixeira and Fernandes, 2014; Teixeira and Gonçalves, 2016). However, some studies show that changes in the functional state of a human operator can also lead to changes in their voice’s F0 and may be a diagnostic sign of significant distress.

### Facial Expressions Analysis

The analysis of the subjects’ facial expressions was carried out using the FaceReader software. The software’s algorithm allows detection of neutral emotional state, as well as six basic emotions according to the generally accepted classification of Ekman and Friesen (1978) (happiness, surprise, sadness, anger, fear, disgust), the general valence of emotions and the level of arousal. The daily video reports were used for the analysis. The FaceReader assesses the general level of a person’s arousal based on the number of facial movements they performs per unit of time. A deep neural network algorithm is used, working in three main stages: detecting a face in the presented image, building a model based on 500 key points and classifying facial expressions (Loijens and Krips, 2018). It is worth noting that a comparison of the expert assessments and the FaceReader software showed its 95% accuracy (Gusev et al., 2018; Stöckli et al., 2018).

### Auxiliary Tests and Questionnaires

The subjects performed computer-aided tests, including psychological questionnaires, twice a day. The State-Trait Anxiety Inventory (STAI) (only the state anxiety was measured) (Spielberger, 2010) and (POMS; Mcnair et al., 1971) questionnaire were used.

Then the subjects performed sensorimotor and cognitive tests simulating operator activity: reaction to a moving object (RMO extrapolation and coordination tasks), short-term memory tests, simple mathematical calculation, response speed (Ushakov et al., 2015).

In the morning after waking up and in the evening before going to bed, medical control was carried out, including measurement of body temperature, heart rate, blood pressure (systolic and diastolic). The data are supplementary to the main research, and are provided by Dr. E.S. Tomilovskaya.

The experimental schedule is presented in Table 1.

**TABLE 1:** Design of the psychophysiological study of female 3-day dry immersion.

Approaches and techniques used	Timing	Duration	Explanations
Medical control	7:30-8 am	5 min	Assessment of body temperature, heart rate, blood pressure (systolic and diastolic).
Speech acoustic analysis	8 am	5 min	F0 (Mean and Median), speech signal intensity, number of pulses, unvoiced speech fragments, jitter and shimmer were analyzed in voice recordings.
Facial expressions analysis			Emotional state assessment using FaceReader software.
**Morning experiments, breakfast**			
State-trait anxiety inventory (STAI)	10 am	1 min	Analysis of the subjective assessment of state anxiety.
Profile of mood states (POMS)		5 min	Analysis of the subjective mood assessment.
Cognitive and sensorimotor tests		10 min	Human operator activity analysis using the computer-aided cognitive and sensorimotor tests.
**Daytime experiments, lunch, rest**			
State-trait anxiety inventory (STAI)	6–7 pm	1 min	Analysis of the subjective assessment of state anxiety.
Profile of mood states (POMS)		5 min	Analysis of the subjective mood assessment.
Cognitive and sensorimotor tests		10 min	Human operator activity analysis using the computer-aided cognitive and sensorimotor tests.
**Dinner, rest**			
Medical control	9 pm	5 min	Assessment of body temperature, heart rate, blood pressure (systolic and diastolic).
Speech acoustic analysis	9–10 pm	5 min	F0 (Mean and Median), speech signal intensity, number of pulses, unvoiced speech fragments, jitter and shimmer were analyzed in voice recordings.
Facial expressions analysis			Emotional state assessment using FaceReader software.

### Statistical Analysis

The used statistical methods included factor analysis by the principal component extraction method (Varimax rotation with Kaiser normalization), correlation analysis (Pearson’s correlation), comparison of paired samples means (Wilcoxon *t*-test), tests of between-subjects effects (ANOVA).

## Results

### Acoustic Analysis of Speech

**FIGURE 1:**
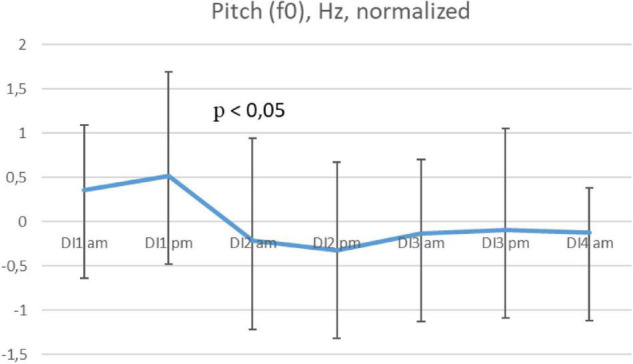
Average mean pitch per morning/evening during the 3-days “dry” immersion experiment. Mean ± SEM.

The values of Mean pitch (fundamental frequency, F0) in the subjects were 205.4 (3.9) Hz on average. The values of Median pitch were 198.4 (3.8) Hz on average. The data was normally distributed. Mean pitch showed a significant decrease by the morning of day 2 in comparison with the evening of the previous day (*p* = 0,04), in 5 out of 6 subjects (Figure 1). A similar result was obtained by Median pitch measurements. In 1 out of 6 subjects, two insignificant decreases in F0 were observed in the evening of the 1st day and in the morning of the 3rd day of the immersion.

**FIGURE 2:**
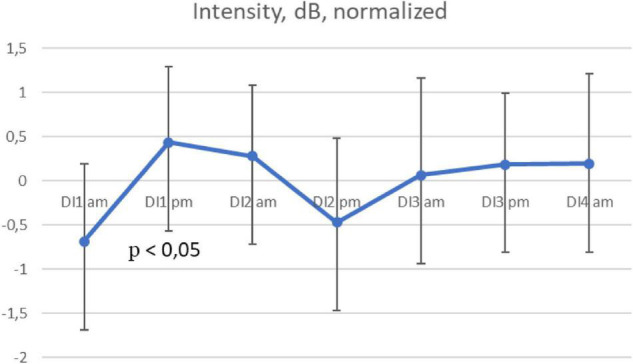
Average intensity per morning/evening during the 3-days “dry” immersion experiment. Mean ± SEM.

The values of speech signal intensity (speech volume) were 80.2 (1.2) dB on average, the data was normally distributed. The speech volume increased significantly by the evening of the day 1 in all subjects (*p* = 0,04) (Figure 2). An insignificant decrease in the speech volume was observed only in the evening of the 2nd day.

### Basic Emotions in Facial Expressions (FaceReader Software)

**FIGURE 3:**
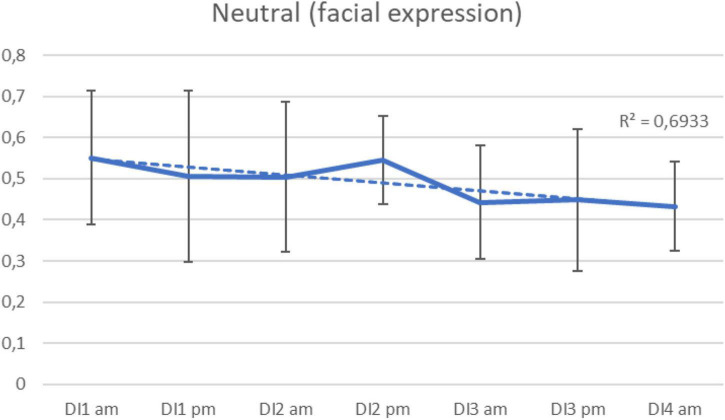
Average neutral emotion (facial expression) per morning/evening during the 3-days “dry” immersion experiment. Mean ± SEM.

Video recording took place simultaneously with speech recording every day, in the morning and in the evening. Based on the results of this study, it was not possible to identify significant trends in basic emotions changes during the immersion. Nevertheless, we were able to identify the dynamics of “neutral” emotional state (neutral facial expression) in the course of the experiment: it gradually decreased (*R*^2^ = 0.7) (Figure 3).

### Psychological Questionnaires

The POMS questionnaire was completed twice a day, in the morning and in the evening, throughout the experiment.

**FIGURE 4:**
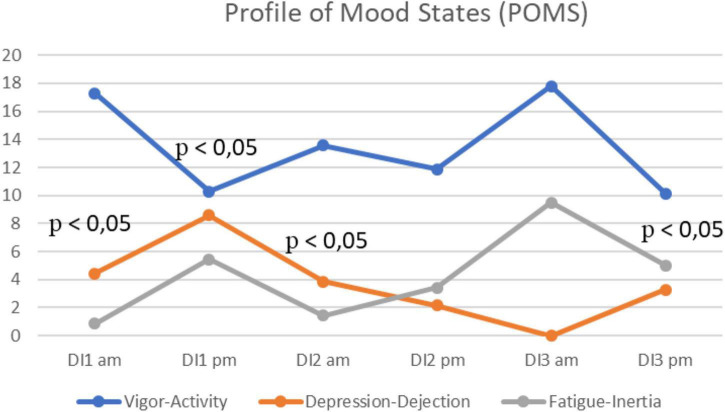
Average mood states (Vigor-Activity, Depression-Dejection and Fatigue-Inertia) per morning/evening during the 3-days “dry” immersion experiment. Mean ± SEM.

In the evening of the 1st day, a sharp decrease in the subjectively perceived Vigor-Activity (*p* = 0.04) and a significant increase in Depression-Dejection (*p* = 0.04) were revealed (Figure 4). By the morning of the 2nd day, the level of Depression-Dejection significantly decreased (*p* = 0.04), as the level of Vigor-Activity slightly increased, while Fatigue decreased (the difference was not statistically significant). On the last day of the experiment, a sharp decrease in Fatigue-Inertia was observed (*p* = 0.04).

**FIGURE 5:**
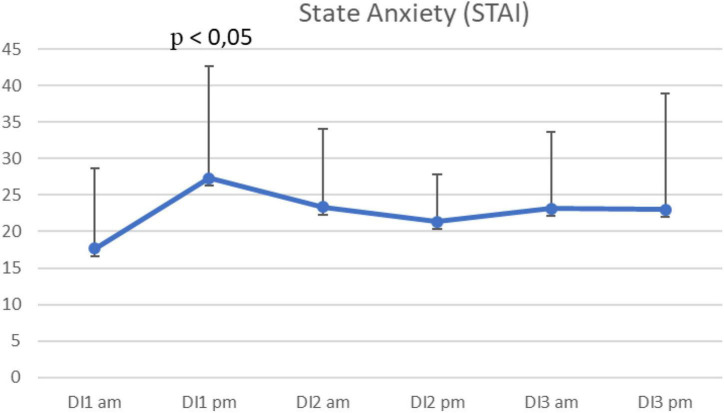
Average state anxiety per morning/evening during the 3-days “dry” immersion experiment. Mean ± SEM.

The state anxiety test according to the STAI questionnaire, presented every day in the morning and in the evening, showed a change in the level of anxiety. The test results can be interpreted as a mild form of S-anxiety (scores less than 30 points) and moderate form of S-anxiety (scores from 31 to 44 points). The peak in the state anxiety was observed in the evening of the first immersion day, which indicates the beginning of the acute phase of adaptation (Figure 5).

### Cognitive and Sensorimotor Tests

Computer tests performed in the morning and evening throughout the DI experiment showed variability in some of the cognitive and sensorimotor functions of the subjects.

**FIGURE 6:**
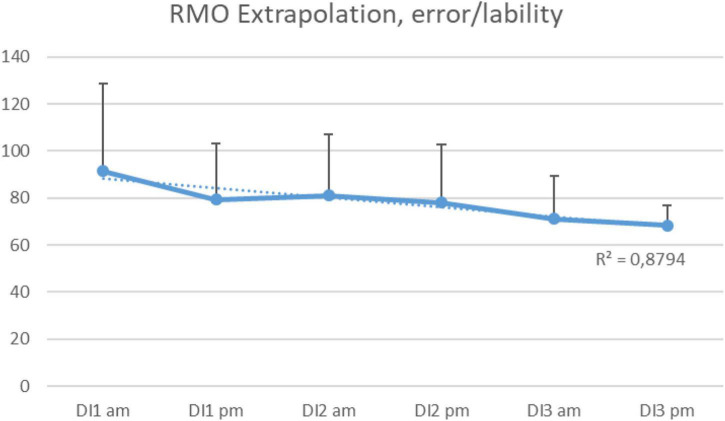
Average error/lability in RMO Extrapolation test per morning/evening during the 3-days “dry” immersion experiment. Mean ± SEM.

The test for sensorimotor coordination “RMO Extrapolation” (Reaction to a Moving Object, motor response to a moving and vanishing point) showed a gradual decrease in error/lability values (Figure 6).

**FIGURE 7:**
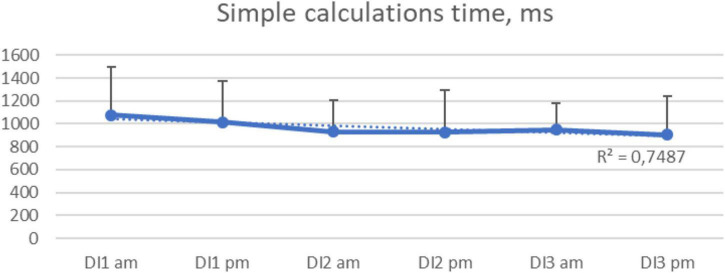
Average time for simple math calculations per morning/evening during the 3-days “dry” immersion experiment. Mean ± SEM.

The time for simple mathematical calculations also decreased during the DI experiment (Figure 7).

### Statistical Analysis

We carried out a factor analysis of data on speech acoustics, the results of cognitive tests, facial expressions, the STAI test results, the POMS questionnaire results and medical control. We used the principal component analysis (rotation method: Varimax with Kaiser normalization. Rotation converged in 12 iterations). The analysis made it possible to identify the components that are significant for this study.

The first component of the resulting matrix shows an inverse relationship between the level of speech intensity and the percentage of pauses, decision time when math calculation, anxiety, hostility, depression, fatigue, anxiety and confusion, and a direct relationship with systolic blood pressure (SBP), activity and friendliness. This component can be described as a state in which a person speaks in a quieter voice, pauses more, solves mathematical equations more slowly, and notes anxiety, hostility, depression, fatigue and confusion, while their SBP is low. The flip side of this component describes a more active and friendly person, with a higher SBP, solving mathematical equations quickly, speaking in a louder voice and making fewer pauses in speech.

The second component shows the inverse relationship between the speech acoustic characteristics (the number of pulses and jitter) and the results of the RMO Extrapolation sensorimotor test (error/lability values). This component could be described as a state of a person in which they utter fewer sounds and their voice «trembles» less, and at the same time they cope better with sensorimotor tasks that require relying on internal time.

The third component showed the relationship between the facial expression of basic emotions according to the FaceReader software: an inverse relationship between sadness and happiness, valence, and diastolic blood pressure (DBP). This component could be described as a state of a person in which they have a higher DBP, and their facial expressions speak of a state of happiness and vivacity.

To clarify the obtained relationships within the components, we carried out an additional correlation analysis (Pearson’s correlation).

### Speech Acoustic Characteristics and the Results of Cognitive Tests

The study revealed significant negative correlations (*p* < 0.01) between such speech characteristic as intensity and the number of pulses, and error/lability values when performing RMO Extrapolation, as well as the simple math calculations time. Also, significant positive correlations were noted between the number of pauses in speech and error/lability values when performing RMO Extrapolation and RMO Coordination tests (*p* < 0.05) and the simple math calculations time (*p* < 0.0001). There was also a positive correlation between Median pitch and the simple math calculations time (*p* < 0.0001). Jitter was inversely correlated with errors in solving mathematical equations (*p* = 0.01).

### Speech Acoustic Characteristics and the Results of Medical Control

Intensity (*p* = 0.001) and the number of pulses (*p* = 0.04) were positively correlated with systolic BP and shimmer was negatively correlated (*p* = 0.001). Intensity (*p* < 0.0001) and the number of pulses (*p* = 0.002) were positively correlated with diastolic BP, and Median pitch (*p* = 0.02), pauses (*p* = 0.006), and shimmer (*p* = 0.02) were negatively correlated. The number of pulses positively correlated with heart rate (*p* = 0.04).

### Speech Acoustic Characteristics and the Results of the Emotions Analysis by Facial Expressions

The number of pulses negatively correlated with «neutral» facial expression (*p* = 0.02), and the percentage of pauses correlated positively (*p* = 0.01). Shimmer positively correlated with the emotion of fear (*p* = 0.001).

### Speech Acoustic Characteristics and the Results of Self-Assessment Questionnaires

#### The Level of State Anxiety

The state anxiety level was positively correlated with Median pitch (*p* = 0.005) and the percentage of pauses (*p* = 0.006), and the number of pulses (*p* = 0.04) was negatively correlated.

#### Profile of Mood States

Median pitch (*p* = 0.01 and *p* = 0.005) and the number of pauses (*p* = 0.01 and *p* = 0.005) correlated with the “hostility” and “depression” scales. Pauses correlated with the fatigue scale (*p* < 0.05). Intensity (*p* = 0.02) and the number of pulses (*p* = 0.007 and *p* = 0.003) were correlated with the “activity” and “friendliness” scales, and pauses (*p* = 0.008 and *p* < 0.0001) were negatively correlated; in addition, Median pitch (*p* = 0.008) was negatively correlated with “friendliness”. Median pitch (*p* = 0.002 and *p* = 0.003) and pauses (*p* = 0.003 and *p* = 0.001) positively correlated with the scales “anxiety” and “confusion”, and in addition, the number of pulses (*p* < 0, 0001).

### Analysis of Basic Emotions by Facial Expressions

Despite the fact that this study did not show a significant number of correlations between basic emotions and data from other methods, we conducted tests of between-subjects effects. The results are presented in Table 2.

**TABLE 2:** Tests of between-subjects effects with basic emotions and days covariates.

Dependent variable:	“RMO extrapolation” error/lability	Time for math calculations	Median pitch	Jitter
Source	Sig.	Sig.	Sig.	Sig.
Corrected model	0,000	0,000	0,011	0,062
Intercept	0,000	0,007	0,004	0,666
N day	0,003	0,119	0,446	0,079
Neutral	0,002	0,052	0,568	0,073
Happy	0,026	0,088	0,685	0,036
Sad	0,160	0,139	0,725	0,043
Angry	0,187	0,120	0,046	0,037
Surprised	0,023	0,052	0,905	0,174
Scared	0,120	0,471	0,588	0,091
Disgusted	0,116	0,134	0,857	0,048
Valence	0,059	0,101	0,541	0,037
Arousal	0,050	0,350	0,029	0,274
Subject name	0,000	0,000	0,004	0,010
Am/Pm	0,323	0,119	0,230	0,475
Subject name Am/Pm	0,030	0,288	0,016	0,397
R squared	0,949	0,960	0,883	0,747
Adjusted R squared	0,852	0,884	0,658	0,263

He showed the influence of such factors as the person of the subject, the number of the day, morning or evening, as well as basic emotions according to FaceReader on the results of cognitive and sensorimotor tests and some acoustic parameters. The results of the analysis show that the person of the subject had the greatest influence, and in some cases, it depended on the time of day (morning or evening). The results of the RMO Extrapolation test were also influenced by the number of the day (which correlates with *R*^2^ for these values).

“Neutral” emotional state influenced the results of RMO Extrapolation and simple calculations. State of “Happiness” influenced the results of RMO Extrapolation and jitter. Jitter was also affected by “Sad” and “Disgusted” emotions. “Surprised” state affected accuracy in RMO Extrapolation and simple calculations.

“Arousal” influenced the results of the RMO Extrapolation and the Median pitch. “Valence” influenced the results of RMO Extrapolation and jitter.

## Discussion

According to the results of our study, the acute period of adaptation in women under simulated microgravity began in the evening of the 1st day and ended in the morning of the 2nd day of immersion. This is indicated by the results of the speech acoustic analysis and self-assessment questionnaires, in which the subjects noted an increase in anxiety and depression, and a decrease in activity. This was compared with the data obtained in the same experiment regarding the attitude of the subjects to their physical discomfort (such as general discomfort, back pain and abdominal pain) also peaking at the 1st day and decreasing by the 2nd day evening (Tomilovskaya et al., 2021). Previously, in studies with participation of astronauts (58% of whom were female) it was shown that approximately half of all astronauts report back pain during the early stages of spaceflight: back pain was most often reported on the first and 2nd days of space flight (Kerstman et al., 2012).

The presence of an acute adaptation period was indicated by a change in the pitch and intensity of the subjects’ voices, which confirms the importance of these non-verbal characteristics of speech for assessing the psychophysiological state of a person. It should be noted that the dynamics of changes in pitch, when compared with the same period of adaptation in men, was less pronounced. In connection with stress, Mean and Median pitch in women significantly increased by the evening of the 1st day in 5 out of 6 subjects, and then decreased to the initial values, and did not show significant fluctuations as it was shown in men under similar conditions. Judging by this indicator, psychophysiological stress in the process of adaptation to microgravity conditions lasted less in women – in a similar experiment, the acute adaptation period in men ended on immersion day 3 (F0 and the number of pulses decreased by the 3rd day) (Lebedeva et al., 2020).

Similar results in terms of sex differences in stress responses were reported previously. A stronger increase in cortisol levels in response to stress in men than in women was shown in some studies (Kudielka and Kirschbaum, 2005). This physiological phenomenon can also cause a stronger increase in some acoustic parameters of the subjects’ speech (Buchanan et al., 2014; Alberdi et al., 2016; Pisanski et al., 2018). At the same time, changes in the subjects’ voice intensity in our study turned out to be almost identical with the same indicator dynamics in male subject during the same period of acute adaptation.

Cognitive tests performed in the morning and evening throughout the experiment showed variability in some mental and sensorimotor functions. The “RMO Extrapolation” test showed a gradual decrease in error/lability values, which correlates with the results of sensorimotor tests obtained from male subjects in longer experiments with “dry” immersion (Lebedeva et al., 2020). The time of simple math equations solving also decreased significantly. We suppose that the observed trends were influenced both by the training effect and stress level decrease. Some authors note that lower quality of sensorimotor and calculation tasks results under simulated (head-down bed rest, parabolic flight) and real space flight conditions may be caused by additional stress associated with increased cognitive load (Wollseiffen et al., 2016; Oluwafemi et al., 2021). Other studies have also shown that “dual-tasking” costs increase for reaction-time tasks requiring rhythm production, and to a lesser degree, visuo-spatial transformation, compared with regular choice reaction-time tasks (Bock et al., 2010). However, in the present study we found no significant difference in performance improvement in two types of tasks: sensorimotor (RMO) and cognitive (mathematical computation), although the sensorimotor task performance is more consistent with the dual-task paradigm than the computation. It is necessary to conduct further studies of longer duration in order to find out the dynamics of these performance changes, and the factors influencing it.

It should be noted that a similar relationship was found between the acoustic characteristics of speech and the sensorimotor performance in the experiment with female subjects and in a similar experiment with men: higher speech volume and the number of pulses were associated with more accurate performance of sensorimotor tasks. This was probably due to an activation tendency in the subjects’ functional state (Cohen et al., 2016; Abur et al., 2021). A significant difference between the female and male samples was a positive (rather than negative, as in men) correlation between the number of pauses in speech and the time of mathematical equations solving. We assume that this distinction needs additional research. Pauses in speech may be interpreted as a sign of concentration, self-control – a transition to the inner voice in order to articulate certain attitudes and rules (Tullett and Inzlicht, 2010). We should also note that the female subjects participated in a 3-day experiment, while the male subjects participated in a 21-day study. Different duration of the “dry” immersion can by itself explain the possible difference in the reasons why the subjects used self-control. In the short experiment with women, the time of mathematical computation was decreasing, as also were the subjects’ anxiety and depression indicators (according to STAI and POMS). It can be assumed that the larger percentage of speech pauses and the longer time spent on mathematical computation were both associated with the subjects’ physical unwellness in the 1st days of adaptation, and, subsequently, with the increased need for self-control (in order to fulfill their tasks despite their current state). We suppose that the reason that female subjects made more pauses in speech during their first audio reports could be that they made additional efforts for self-regulation, and were also more worried about the correct task results, so they additionally used the inner voice to recite the rules of the task (Laukka et al., 2008). At the same time, the male sample, while solving the computation task at a much later period of the experiment, probably used the inner voice to decelerate impulsive responding. I.e., on the days when the male subjects felt more disinhibited (and, subsequently, spoke faster, with less pauses), they tried to take more time for better computation performance, slowing down their motor reaction (pressing a key to respond) (Tullett and Inzlicht, 2010).

Also, the distinction of the female sample were the significant correlations between objective psychophysiological indicators (acoustic analysis of speech and analysis of facial expressions) and the results of self-assessment tests. The results of self-assessment questionnaires correlate well with each other and with the data of acoustic analysis of speech. This indicates that the subjects in the present study interpreted their psychological state well and answered honestly to the questionnaire questions.

The method of the basic emotions analysis using facial expressions revealed some limitations of this study: the body position (recumbent), as well as the redistribution of body fluids in the cranial direction, could affect the quality of the analysis. The most informative was the indicator of “neutral” emotional state – during the 3-day exposure, it significantly decreased. It should be noted that “neutral” emotional states positively correlated with the percentage of pauses in speech and negatively – with vocal pulses. A similar pattern was found between the same acoustic indicators and the subjects’ perception of anxiety (STAI and POMS) and confusion (POMS). This can be interpreted as a mild variant of a “freeze” stress reaction, when a person speaks less, their face becomes less emotional, and at the same time they feel anxious and confused. Some studies have noted similar reactions in connection with social stress (social phobias): under the influence of increasing anxiety, the percentage of pauses in voluntary speech increases (Laukka et al., 2008; Buchanan et al., 2014).

## Conclusion

The conducted psychophysiological study revealed some indicators of the acute period of adaptation to microgravity conditions. For the first time conducted with the participation of female subjects, this study showed possible differences from all-male samples: in the duration and specificity of adaptation to «dry» immersion conditions, in a more accurate reflection of one’s state in questionnaires, as well as in some patterns of correlations between the acoustic characteristics of speech and the results of cognitive tests.

The acute adaptation period in female subjects began earlier and ended faster than in male ones in previous studies. This was indicated by a decrease in the level of state anxiety (STAI) and depression-dejection (POMS), as well as a decrease in pitch and voice intensity. Women in this study reacted to the adaptation stress differently than men previously: a significant decrease in pitch was shown, while in men under the same conditions the opposite was more often observed. In addition, women, apparently, used the “freeze” coping strategy – the proportion of neutral facial expressions on the most intense days of the experiment was at maximum. Thus, the subjects in this experiment understood their feelings and emotions better, giving more accurate answers in self-assessment questionnaires, but at the same time tried to look and sound as calm and confident as possible, controlling their expressions.

Same trends in the subjects’ cognitive performance were identified as in similar experimental conditions earlier: an excited state (high intensity and a higher number of pulses in speech) corresponded to better performance of sensorimotor tasks. The difference was in the speed of mathematical computation: women in the present study performed the computation faster on the same days when they made fewer pauses in speech (i.e., spoke more quickly), while in men in previous experiments this relationship was inverse: on the days when male subjects made more pauses in speech, they performed the computation faster.

The results obtained need to be corroborated both in the «dry» immersion model and in other physiologically stressful conditions. In studies related to speech, facial expressions and cognitive performance, it is necessary to take into account the subjects’ individual differences, and also, the specific influence of the experimental conditions and the method of obtaining experimental data.

In order to get a better understanding of the influence of physiologically stressful conditions on the human operator’s psychophysiological state, further studies with the participation of women are required.

## Data Availability Statement

The raw data supporting the conclusions of this article will be made available by the authors, without undue reservation.

## Ethics Statement

The studies involving human participants were reviewed and approved by Bioethical Commission of the Institute of Biomedical Problems of RAS. The patients/participants provided their written informed consent to participate in this study.

## Author Contributions

SL prepared the analysis of acoustic speech characteristic, sensorimotor cognitive tests and psychological questionnaire, and wrote the draft of the manuscript. AS prepared the analysis of FaceReader data. DS made the manuscript revisions. All authors contributed to the article and approved the submitted version.

## Conflict of Interest

The authors declare that the research was conducted in the absence of any commercial or financial relationships that could be construed as a potential conflict of interest.

## Publisher’s Note

All claims expressed in this article are solely those of the authors and do not necessarily represent those of their affiliated organizations, or those of the publisher, the editors and the reviewers. Any product that may be evaluated in this article, or claim that may be made by its manufacturer, is not guaranteed or endorsed by the publisher.

## References

[B1] Abur D. MacPherson M. K. Shembel A. C. Stepp C. E. (2021). Acoustic Measures of Voice and Physiologic Measures of Autonomic Arousal During Speech as a Function of Cognitive Load in Older Adults. *J. Voice* 2021:27. 10.1016/j.jvoice.2020.12.027 33509665 PMC8310524

[B2] Akçay M. B. Oguz K. (2020). Speech emotion recognition: Emotional models, databases, features, preprocessing methods, supporting modalities, and classifiers. *Speech Comm.* 116 56–76. 10.1016/j.specom.2019.12.001

[B3] Alberdi A. Aztiria A. Basarab A. (2016). Towards an automatic early stress recognition system for office environments based on multimodal measurements: a review. *J. Biomed. Inform.* 59 49–75. 10.1016/j.jbi.2015.11.007 26621099

[B4] Bock O. Weigelt C. Bloomberg J. J. (2010). Cognitive demand of human sensorimotor performance during an extended space mission: a dual-task study. *Aviat. Space Environ. Med.* 81 819–824. 10.3357/asem.2608.2010 20824987

[B5] Boersma P. Weenink D. (2018). *Praat: doing phonetics by computer [Computer program]. Version 6.0.37.* Available online at: from http://www.praat.org (accessed date 14 March 2018)

[B6] Buchanan T. W. Laures-Gore J. S. Duff M. C. (2014). Acute stress reduces speech fluency. *Biol. Psychol.* 97 60–66. 10.1016/j.biopsycho.2014.02.005 24555989

[B7] Cermack M. (2006). Monitoring and telemedicine support in remote environments and in human space flight. *Br. J. Anaesth.* 97 107–114. 10.1093/bja/ael132 16731572

[B8] Cohen A. O. Dellarco D. V. Breiner K. Helion C. Heller A. S. Rahdar A. (2016). The Impact of Emotional States on Cognitive Control Circuitry and Function. *J. Cogn. Neurosci.* 28 446–459. 10.1162/jocn_a_00906 26601909

[B9] Egorov A. D. (2001). Theory and methodology of medical control in long space flights. Official speech. RAS. SSC RF – IBMP RAS. Moscow. Available online at: http://www.imbp.ru/webpages/win1251/science/Egorov_actsp.html (accessed January 5, 2022).

[B10] Ekman P. Friesen W. V. (1978). *Manual for the facial action coding system.* Palo Alto: Consulting Psychologists Press. 10.1037/t27734-000

[B11] Fontes M. Madureira S. (2019). “Vocal and facial expressions and meaning effects in speech expressivity,” in *Proceedings of 10th International Conference on Experimental Linguistics, 25-27 September* (Lisbon). 10.36505/ExLing-2019/10/0020/000382

[B12] Giddens C. L. Barron K. W. Byrd-Craven J. Clark K. F. Winter A. S. (2013). Vocal indices of stress: a review. *J. Voice* 27 .e21–.e390. 10.1016/j.jvoice.2012.12.010 23462686

[B13] Gusev A. N. Engalychev V. F. Zakharova N. A. (2018). “Modern trends in the use of software and hardware in assessing the psychoemotional state of a person,” in *Hardware in psychological training (in Russian)*, eds Karayani A. G. Danilov S. I. (New York, NY: Military University), 110–117.

[B14] Gushchin V. I. Savinkina A. O. Shved D. M. (2020). “Analysis of the emotional state participants in a 4-month isolation experiment using the assessment method facial expressions FaceReader,” in *Abstracts of the IV International Scientific practical conference “Topical issues of forensic psychological examination and comprehensive examination with the participation of a psychologist. Modern computer technologies in expert practice”. December 18-19* (Kaluga), 9.

[B15] Gushchin V. I. Vinokhodova A. G. Komissarova D. V. Belakovsky M. S. Orlov O. I. (2018). Experiments with isolation: the past, present and future. *Aviak. Ekologic. Med.* 4:5. 10.21687/0233-528X-2018-52-4-5-16

[B16] Gushin V. I. Yusupova A. K. Shved D. M. Shueva L. V. Vinokhodova A. Bubeev Y. (2016). The evolution of methodological approaches to the psychological analysis of the crew communications with Mission Control Center. *REACH – Rev. Hum. Space Explorat.* 1 74–83. 10.1016/j.reach.2016.05.001

[B17] Johannes B. Salnitski V. P. Gunga H. C. Kirsch K. (2000). Voice stress monitoring in space: possibilities and limits. *Aviat Space Environ Med.* 71(9 Suppl.), 58–65. 10993311

[B18] Johannes B. Salnitski V. Soll H. Rauch M. Hoermann H. (2008). Deindividualized psychophysiological strain assessment during a flight simulation test – validation of a space methodology. *Acta Astronaut.* 63 791–799.

[B19] Johannes B. Wittels P. Enne R. Eisinger G. Castro C. A. Thomas J. L. (2007). Non-linear function model of voice pitch dependency on physical and mental load. *Eur. J. Appl. Physiol.* 101 267–276. 10.1007/s00421-007-0496-6 17554549

[B20] Kartavenko M. V. (2005). About the use of acoustic characteristics of speech for the diagnosis of human mental states. *Izvestiya YuFU. Tekhnicheskie nauki* 2005:5.

[B21] Kerstman E. L. Scheuring R. A. Barnes M. G. DeKorse T. B. Saile L. G. (2012). Space adaptation back pain: a retrospective study. *Aviat. Space Environ. Med.* 83 2–7. 10.3357/ASEM.2876.2012 22272509

[B22] Kudielka B. M. Kirschbaum C. (2005). Sex differences in HPA axis responses to stress: a review. *Biol. Psychol.* 69 113–132. 10.1016/j.biopsycho.2004.11.009 15740829

[B23] Laukka P. Linnman C. Ahs F. Pissiota A. Fran O. Faria V. (2008). In a nervous voice: Acoustic analysis and perception of anxiety in social phobics’ speech. *J. Nonverb. Behav.* 32 195–214. 10.1007/s10919-008-0055-9

[B24] Lebedeva S. A. Shved D. M. Fedyai S. O. (2020). Investigation of human psychophysiological state in the conditions simulating the microgravity effects using the acoustic method of speech analysis. *Aviakosmicheskaya i Ekologicheskaya Meditsina* 54 45–51. 10.21687/0233-528x-2020-54-2-45-51

[B25] Lebedeva S. A. Shved D. M. (2021). Comparison of the human psychophysiological status in the conditions of simulated microgravity without countermeasures and during rotation on a short-arm centrifuge. *Aviakosmicheskaya i Ekologicheskaya Meditsina* 55 98–101. 10.21687/0233-528x-2021-55-2-98-101

[B26] Loijens L. Krips O. (2018). *FaceReader Methodology Note.* Netherlands: Behavioral research consultants at Noldus Information Technology.

[B27] Mcnair D. M. Lorr M. Droppleman L. F. (1971). *Manual for the profile of mood states.* San. Diego, CA: Educational and Industrial Testing Services.

[B28] Mikhailov V. M. Reushkin V. N. Reushkina G. D. Sebekina T. V. Smirnova T. M. (1995). Using variance analysis for the influence of immersion and individuality on the variability of orthostatic reactions. *Aviakosm Ekolog Med.* 6 26–32. 8664883

[B29] National Aeronautics and Space Administration and the National Center for Gender Physiology and Environmental Adaptation (2002). *Sex, Space and Environmental Adaptation: A National Workshop on Research Priorities on Sex Differences in Human Responses to Challenging Environments.* Available online at: https://www.nasa.gov/pdf/185051main_environmental_adaptation_workshop_11-2002.pdf (accessed June 31, 2021).

[B30] Nikonov A. V. (1985). *Psychological problems of acoustic diagnostics of functional states of an operator.* Moscow: Psychological problems of activity in special conditions, 136–153.

[B31] Oluwafemi F. A. Abdelbaki R. Lai J. C.-Y. Mora-Almanza J. G. Afolayan E. M. (2021). A review of astronaut mental health in manned missions: Potential interventions for cognitive and mental health challenges. *Life Sci. Space Res.* 28 26–31. 10.1016/j.lssr.2020.12.002 33612177

[B32] Orlov V. N. (1985). Influence of Dry Immersion model on the performance of water-salt exchange, aldosterone and cortisol level in plasma in patients with different degree of hydration of the body. *Space Biol. Aerospace Med.* 4 42–45.

[B33] Park Y. Stepp C. E. (2019). The Effects of Stress Type, Vowel Identity, Baseline f0, and Loudness on the Relative Fundamental Frequency of Individuals With Healthy Voices. *J. Voice* 33 603–610. 10.1016/j.jvoice.2018.04.004 30078521 PMC6557681

[B34] Pisanski K. Aleksander K. Luba J. Judyta N. Amelia W. Kamil B. (2018). Multimodal stress detection: testing for covariation in vocal, hormonal and physiological responses to Trier Social Stress Test. *Horm Behav.* 106 52–61. 10.1016/j.yhbeh.2018.08.014 30189213

[B35] Rothkrantz J. M. (2004). “Voice stress analysis,” in *Conference Paper in Lecture Notes in Computer Science* (Springer Science+Business Media).

[B36] Sanchez K. Oates J. Dacakis G. (2014). Speech and voice range profilesof adults with untrained normal voices: Methodological implications. *Logop Phoniatr. Vocol.* 39 62–71. 10.3109/14015439.2013.777109 23590284

[B37] Saralyn M. (2006). The impact of sex and gender on human adaptation to space. *Gend. Med. Vol.* 3:22.

[B38] Shul’zhenko E. B. Vill-Villiams I. F. (1976). Possibility of carrying outprolonged water immersion by the method of “dry” immersion (in Russian). *Kosm. Biol. Aviakosm. Med.* 10 82–84. 1263423

[B39] Spielberger C. D. (2010). *State-trait anxiety inventory. The Corsini encyclopedia of psychology.* Hoboken, NJ: John Wiley & Sons, Inc. 10.1002/9780470479216.corpsy0943

[B40] Stöckli S. Schulte-Mecklenbeck M. Borer S. Samson A. C. (2018). Facial expression analysis with AFFDEX and FACET: A validation study. *Behav. Res. Methods. V.* 50 1446–1460. 10.3758/s13428-017-0996-1 29218587

[B41] Supolkina N. S. Yusupova A. K. Shved D. M. Chekalina A. I. Sarantsev S. V. Gushchin V. I. (2019). Communication behavior of the crew when communicating with the control center in experiment sirius-17. *Aerosp. Env. Med.* 53 68–73. 10.21687/0233-528X-2019-53-2-68-73

[B42] Teixeira J. P. Fernandes P. O. (2014). Jitter, Shimmer and HNR classification within gender, tones and vowels in healthy voices. *Proced.* T*ech.* 16 1228–1237. 10.1016/j.protcy.2014.10.138

[B43] Teixeira J. P. Gonçalves A. (2016). Algorithm for jitter and shimmer measurement in pathologic voices. *Proced. Comp. Sci.* 100 271–279. 10.1016/j.procs.2016.09.155

[B44] Teixeira J. P. Ferreira D. Carneiro S. (2011). “Análise acústica vocal - determinação do Jitter e Shimmer para diagnóstico de patalogias da fala,” in *6° Congresso Luso-Moçambicano de Engenharia* (Maputo).

[B45] Tomilovskaya E. Amirova L. Nosikova I. Rukavishnikov I. Chernogorov R. Lebedeva S. (2021). The First Female Dry Immersion (NAIAD-2020): Design and Specifics of a 3-Day Study. *Front. Physiol.* 12:284. 10.3389/fphys.2021.661959 34194336 PMC8236811

[B46] Tomilovskaya E. Shigueva T. Sayenko D. Rukavishnikov I. Kozlovskaya I. (2019). Dry Immersion as a Ground-Based Model of Microgravity Physiological Effects. *Front. Physiol.* 10:284. 10.3389/fphys.2019.00284 30971938 PMC6446883

[B47] Tullett A. M. Inzlicht M. (2010). The voice of self-control: Blocking the inner voice increases impulsive responding. *Acta Psychol.* 135 252–256. 10.1016/j.actpsy.2010.07.008 20692639

[B48] Ushakov I. B. Ivanov A. V. Kvasovets S. V. Bubeev Yu A. (2015). Neurosemantic and psychophysiological correlates of rhythm-suggestive correction of stress conditions. *Aviakosmicheskaya i ekologicheskaya meditsina* 49 55–60. 26934791

[B49] Wollseiffen P. Vogt T. Abeln V. Strüder H. K. Askew C. D. Schneider S. (2016). Neuro-cognitive performance is enhanced during short-periods of microgravity. *Physiol. Behav.* 155 9–16. 10.1016/j.physbeh.2015.11.036 26657021

[B50] Zhang Z. (2016). Mechanics of human voice production and control. *J. Acoust. Soc. Am.* 140:2614. 10.1121/1.4964509 27794319 PMC5412481

[B51] Zwetsch I. Fagundes R. Russomano T. Scolari D. (2006). *Digital signal processing in the differential diagnosis of beningn larynx diseases.* Porto Alegre: Directory of Open Access Journals.

